# The correlation of urinary strontium with the risk of chronic kidney disease among the general United States population

**DOI:** 10.3389/fpubh.2023.1251232

**Published:** 2023-09-13

**Authors:** Fenglian Zhang, Na Hu, Jiayue Li, Ming Pu, Xinchun Li, Yuanmei Li, Dan Liao

**Affiliations:** ^1^Department of Nephrology, Mianyang Central Hospital, School of Medicine, University of Electronic Science and Technology of China, Mianyang, China; ^2^Chengdu Medical College, Chengdu, China; ^3^North Sichuan Medical College, Nanchong, China

**Keywords:** chronic kidney disease, heavy metal, kidney function, NHANES, urinary strontium

## Abstract

**Background:**

This study sought to illustrate whether urinary strontium levels were related to developing chronic kidney disease (CKD) in the United States population.

**Methods:**

A total of 5,005 subjects were identified from the National Health and Nutrition Examination Survey 2011–2016. Survey-weighted logistic regression analysis, multivariate linear regression analysis, restricted cubic spline (RCS) plots curve and stratified analyses were undertaken to explicate the correlation between urinary strontium and CKD.

**Results:**

With the increase of urinary strontium, the incidence rate of CKD and urinary albumin to creatinine ratio (UACR) levels gradually decreased, and estimated glomerular filtration rate (eGFR) levels gradually increased. After controlling all confounders, only urinary strontium in the fourth quartile was correlated to a lower CKD prevalence (OR: 0.59; 95% CI, 0.44–0.79) compared to the lowest quartile. Multivariate linear regression analysis showed that urinary strontium was positively correlated with eGFR but negatively with UACR. RCS curve suggested a nonlinear relationship between urinary strontium and CKD (*P* for non-linearity <0.001). Stratified analyses indicated no significant difference in the correlation between urinary strontium and CKD among different subgroups.

**Conclusion:**

Urinary strontium was strongly correlated with a low risk of CKD, and this association was non-linear among the US population.

## 1. Introduction

Chronic kidney disease (CKD) is a significant health dilemma and causes substantial healthcare expenditure. The end-stage renal disease prevalence in the United States (US) remains one of the highest in the world (1). According to the US Renal Data System Annual Data Report, the CKD prevalence of US adults had been relatively stable at just under 15% for the last 15 years, which was likely caused by the high prevalence of aging populations, diabetes, hypertension, obesity, cardiovascular disease, and other conditions (1, 2). But the known etiologies do not cause all CKD (3). Some metals, including cadmium, lead, and chromium, accumulated in the kidneys may harm renal function (4–6).

Strontium is an alkaline earth metal considered a trace element in the human bone, the only one that is correlated with bone compression strength (7, 8). Strontium is primarily derived from food and beverages, with strontium content in grains and seafood reaching up to 25 mg/kg (9). Strontium is absorbed through the gastrointestinal tract, mainly stored in bones and blood, and excreted through the kidneys (10). Thus, patients with renal dysfunction are at an increased risk of accumulating this metal (9). Prior researches have similarly indicated that dialysis patients had higher serum strontium accumulation than healthy individuals (11, 12). Subsequent findings have further showed that elevated levels of serum strontium pose a risk of kidney toxicity. For example, researchers suggested that serum strontium was associated with an increased risk of renal insufficiency (13, 14). Besides, serum strontium has been proven to be negatively related to the estimated glomerular filtration rate (eGFR) in the diabetic population (15). Another study showed that patients with high serum strontium were at an increased risk for acute kidney injury after cardiac surgery (16). In fact, urine is more convenient and less invasive than blood. However, the available evidence on the relationship between urinary strontium and CKD is limited. A study conducted on the population of Netherlands regarding diabetic kidney disease revealed a decrease in urinary strontium as eGFR decreases (15). Another Chinese study reported that urinary strontium was positively correlated with eGFR (17). But those findings did not further explore the correlation between urinary strontium and the risk of CKD. There is no evidence regarding the associations of urinary strontium with CKD in the US general population. The National Health and Nutrition Examination Survey (NHANES) employs rapid and accurate quantitative techniques to examine urine samples obtained from individuals who are potentially exposed to various significant metal elements, or to evaluate environmental or other nonoccupationally exposure to these same elements. Accordingly, we performed a cross-sectional study to examine the relationship of urinary strontium with the prevalence of CKD among the general population based on NHANES.

## 2. Methods

### 2.1. Study participants

**Figure 1:**
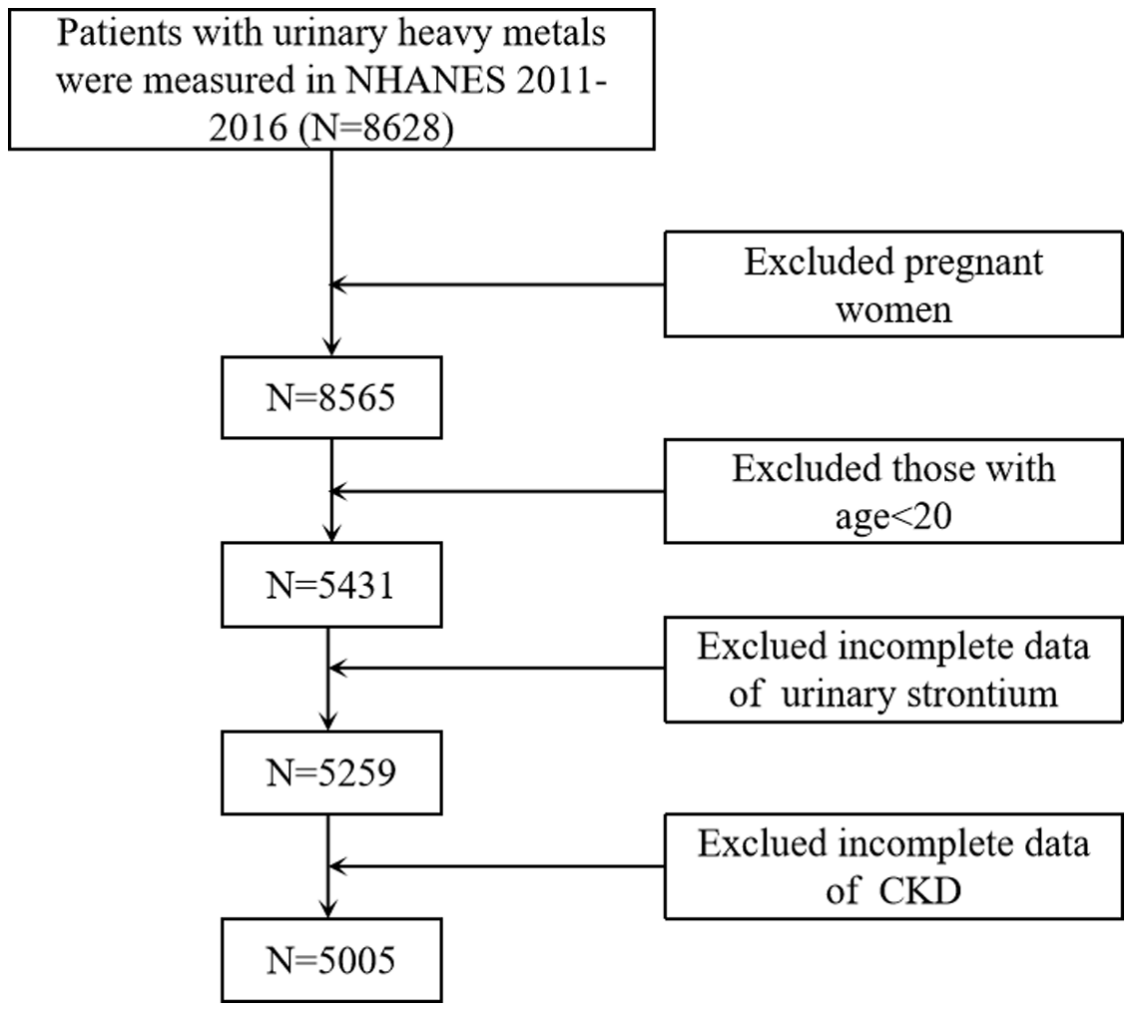
Flowchart of the sample selection from NHANES 2011–2016. NHANES, National Health and Nutrition Examination Surveys; CKD, chronic kidney disease.

Multistage, stratified probability sample designs were used in the NHANES to obtain a representative sample (18). Each subject in NHANES has signed an informed consent form. Approval was acquired from the Research Ethics Review Board of the National Center for Health Statistics. Thus, this paper does not require additional ethical approval. A cross-sectional analysis was conducted using the 2011–12, 2013–14, and 2015–16 (three) data cycles since urine strontium data was available. Those measured for heavy metals in the spot urine samples were confirmed (*n* = 8,628). Pregnant women (*n* = 63), individuals younger than 20 years (*n* = 3,134), and those with incomplete urinary strontium (*n* = 172) or CKD (*n* = 254) were excluded from the final analysis. Finally, 5,005 respondents were selected for this study (Figure 1).

### 2.2. Covariates

Covariates were acquired by trained health technicians through in-person interviews utilizing a standardized questionnaire, physical examinations, and laboratory tests. Demographic characteristics included sociodemographic characteristics [age, sex (male and female), race/ethnicity (black, white, Mexican and others), education levels (less than high school, high school graduates, and above high school), marital status (separated and married), family poverty-to-income ratio (<1.3, 1.3–3.5, and ≥3.5), body mass index (BMI)], lifestyle behaviors [smoking status (never, former, and current), alcohol consumption (none, mild, moderate, heavy, and former), and physical activity (never, moderate, and vigorous)], medical history (hypertension, diabetes, hyperlipidemia, anemia, and hyperuricemia), serum calcium, and phosphorus. BMI was calculated as weight (kg)/height (m) (2). Diabetes was ascertained through self-reported diagnosis, utilization of diabetes medications, a hemoglobin A1c level of 6.5% or higher, or a fasting plasma glucose level of 126 mg/dL or higher (19). Hypertension was diagnosed based on self-reported diagnosis, utilization of anti-hypertension medications, or a systolic/diastolic blood pressure reading of 140/90 mmHg or higher (20). Hyperlipidemia was determined by self-reported diagnosis, a triglyceride level of 200 mg/dL or higher, a high-density lipoprotein cholesterol level below 40 mg/dL, or a low-density lipoprotein cholesterol level of 130 mg/dL or higher (21). In accordance with established criteria, anemia was defined as a hemoglobin concentration below 13 g/dL in males and below 12 g/dL in females (22). Similarly, hyperuricemia was considered as a serum urate level equal to or exceeding 7.0 mg/dL in men and equal to or exceeding 6 mg/dL in women (23).

### 2.3. Quantification of strontium in urine

Urine samples were processed, stored, and transported to the Laboratory Science Department, National Center for Environmental Health, Centers for Disease Control and Prevention for subsequent analysis. Inductively coupled plasma-mass spectrometry was employed to analyze urine strontium. Comprehensive information about specimen collection, processing instructions, and quality control can be found in the NHANES Laboratory Procedures Manual. Besides, the data underwent a thorough review process, whereby incomplete data or values deemed improbable were forwarded to the performing laboratory for confirmation. In the case of analytic results below the lower detection limit, this value was filled in the lower detection limit divided by the square root of 2. A cohort of 5,005 participants were divided into four quartiles: namely the first quartile (Q1) (1.768–48.76 μg/L), the second quartile (Q2) (48.76–89.43 μg/L), the third quartile (Q3) (89.43–154.05 μg/L), and the fourth quartile (Q4) (>154.05 μg/L), to evaluate the association between urinary strontium and CKD. The reference group was created using Q1.

### 2.4. Assessment of CKD

CKD is defined as an eGFR <60 mL/min/1.73 m^2^ and/or urinary albumin to creatinine ratio (UACR) ≥30 mg/g (24, 25). According to the eGFR (G1–G5) and proteinuria category (A1–A3), the prognosis of CKD was stratified as low risk, medium risk, high risk, and very high risk (26, 27). The eGFR was computed through the CKD-Epidemiology Collaboration equation, which includes serum creatinine, age, sex, and ethnicity (28, 29). The calculation for eGFR was as follows: 141 × min (SCr/*κ*, 1)*^α^* × max (SCr/*κ*, 1)^−1.209^ × 0.993^Age^ × 1.018 if female × 1.159 if black, where SCr represents serum creatinine, *κ* is 0.7 for females and 0.9 for males, *α* is −0.329 for females and −0.411 for males, min denotes the lesser value between SCr/*κ* or 1, and max signifies the greater value between SCr/*κ* or 1.

### 2.5. Statistical analysis

We used appropriate sample weights and strata in all analyses because of NHANES’s complex sampling design (30). Continuous data were calculated using the survey-weighted mean ± standard error and tested using the one-way ANOVA. Categorical variables were expressed as survey-weighted percentages and compared using the survey-weighted Chi-square test. The association between urinary strontium and CKD prevalence was evaluated using survey-weighted logistic regression analysis and performed in three different models. Model I was a crude model and none of the covariates were included. In model II, the selection of potential confounders was based on the criterion of changes in beta coefficients for our primary exposure exceeding 10%. Thus, we adjusted for age, education level, family poverty-to-income ratio, smoking status, hypertension, diabetes, anemia, serum calcium, and phosphorus. Model III was a fully adjusted model and included age, sex, race/ethnicity, BMI, education level, marital status, family poverty-to-income ratio, smoking status, alcohol consumption, physical activity, hypertension, diabetes, hyperlipidemia, anemia, hyperuricemia, serum calcium, and phosphorus. Survey-weighted multivariate linear regression analysis was implemented to determine the association between urinary strontium and kidney biomarkers (eGFR and UACR). UACR was transformed into log_2_ to improve the normality due to a skewed distribution (31). Restricted cubic spline (RCS) regression was used to detect whether there was a linear correlation between urinary strontium and CKD prevalence (32, 33). The study population was stratified by age, gender, hypertension, hyperlipidemia, and diabetes for conducting subgroup analyses. All analyses were conducted using R software (version 3.6.3). Statistical significance was defined as *p*-values <0.05.

## 3. Results

### 3.1. Characteristics of participants

As shown in Table 1, the average ages of the four different quartiles of urinary strontium were 45.35 (1.02), 46.19 (1.02), 44.72 (1.02), and 42.24 (1.02) years with a significant difference, respectively. With the increase of urinary strontium, a stepwise downward trend was observed in the level of UACR, the prevalence of CKD, and anemia (*p* < 0.05). Besides, a stepwise upward trend was found in eGFR (*p* < 0.05). The prognosis risk of CKD decreased with the increase of urinary strontium. Participants in Q4 had the highest proportion of males and levels of serum calcium (all *p* < 0.05). The family poverty-to-income ratio differed significantly (*p* < 0.05) among the quartiles of urinary strontium (Table 1).

**Table 1:** Participant characteristics in NHANES 2011–2016, weighted.

Variables	Quartile categories of urinary strontium (ug/L)					*p*
	Total	Quartile 1	Quartile 2	Quartile 3	Quartile 4	
		1.768–48.76	48.76–89.43	89.43–154.05	>154.05	
Age (years)	44.60 (1.01)	45.35 (1.02)	46.19 (1.02)	44.72 (1.02)	42.24 (1.02)	<0.001
Sex						<0.001
Male	48.88	39.85	45.56	53.88	56.00	
Female	51.12	60.15	54.44	46.12	44.00	
Race/ethnicity						0.081
Black	10.76	11.62	11.22	11.65	8.54	
White	66.12	66.98	65.72	66.62	65.15	
Mexican	8.64	7.83	9.02	7.96	9.77	
Other	14.48	13.57	14.04	13.77	16.54	
BMI (kg/m^2^)	28.27	27.86	28.09	28.52	28.59	0.174
Education level						0.178
Less than high school	15.17	13.29	16.69	14.49	16.22	
High school graduates	20.61	18.80	19.87	22.33	21.39	
Above high school	64.21	67.91	63.44	63.18	62.40	
Marital status						0.849
Separated	37.33	37.81	37.05	36.20	38.29	
Married	62.66	62.19	62.95	63.80	61.71	
Family poverty-to-income ratio						0.024
<1.3	20.79	22.37	24.10	20.19	23.19	
1.3–3.5	33.06	32.16	32.71	38.85	38.88	
≥3.5	38.83	45.47	43.19	40.96	37.93	
Smoking status						0.254
Never	56.11	60.04	55.49	56.63	52.33	
Former	25.02	22.96	25.99	24.41	26.73	
Current	18.86	16.99	18.52	18.96	20.95	
Alcohol consumption						0.486
None	10.47	10.69	11.84	11.61	11.14	
Mild	33.32	36.50	38.17	35.10	34.44	
Moderate	16.30	19.65	16.95	16.74	17.23	
Heavy	19.59	18.56	19.42	22.25	24.53	
Former	12.75	14.61	13.61	14.30	12.66	
Physical activity						0.075
Never	56.10	58.10	56.47	55.79	54.08	
Moderate	10.02	12.28	8.80	8.56	10.49	
Vigorous	33.87	29.62	34.73	35.64	35.43	
Hypertension	37.90	39.69	40.56	35.84	35.59	0.215
Diabetes	15.21	16.23	16.79	15.17	12.68	0.120
Hyperlipidemia	68.38	67.86	67.94	69.25	68.43	0.931
CKD	14.23	17.65	15.98	13.00	10.39	<0.001
Anemia	6.93	9.06	7.65	6.15	5.12	0.005
Hyperuricemia	18.60	19.33	17.28	17.21	21.17	0.197
Prognosis of CKD						<0.001
Low risk	85.77	83.15	84.95	87.46	90.04	
Moderate risk	9.53	9.09	11.29	10.03	8.00	
High risk	2.58	4.09	2.68	2.03	1.64	
Very high risk	1.36	3.67	1.08	0.48	0.32	
eGFR (mL/min/1.73 m^2^)	91.50 (1.01)	87.90 (1.01)	91.20 (1.01)	92.58 (1.01)	94.35 (1.01)	0.002
UACR (mg/g)	8.70 (1.02)	9.95 (1.04)	9.08 (1.04)	8.00 (1.04)	7.94 (1.03)	<0.001
Serum calcium (mg/dL)	9.40 (1.00)	9.37 (1.00)	9.41 (1.00)	9.39 (1.00)	9.43 (1.00)	0.025
Serum phosphorus (mg/dL)	3.71 (1.00)	3.70 (1.01)	3.74 (1.01)	3.69 (1.01)	3.73 (1.01)	0.197

BMI, body mass index; CKD, chronic kidney disease; eGFR, estimated glomerular filtration rate; UACR, urinary albumin-to-creatinine ratio; NHANES, National Health and Nutrition Examination Surveys.

### 3.2. Association of urinary strontium with CKD

**Figure 2:**
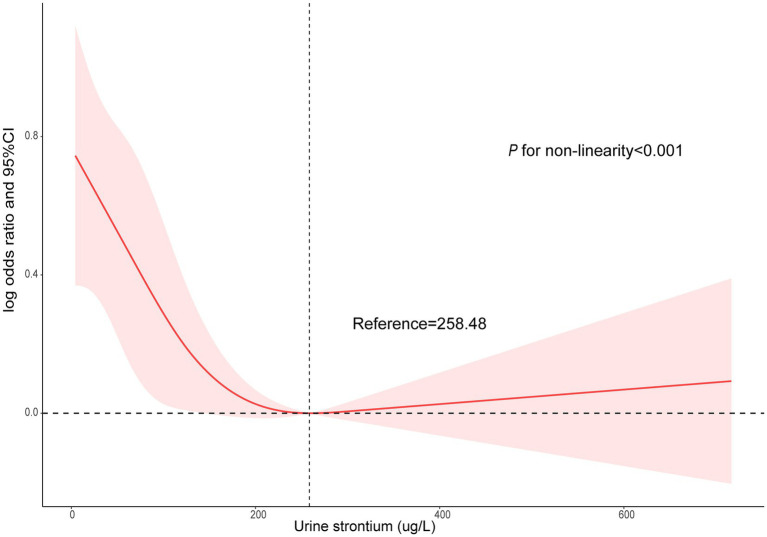
Non-linear association between urinary strontium and CKD by the restricted cubic spline model. CKD, chronic kidney disease.

The survey-weighted logistic regression method was implemented to observe the correlation between urinary strontium and CKD (Table 2). The model I indicated that compared with individuals in Q1, a significantly lower prevalence of CKD was observed in Q3 (OR, 0.70; 95% CI: 0.50, 0.96) and Q4 (OR, 0.54; 95% CI: 0.43, 0.69). In model II, only Q4 was still related to a decreased risk of CKD relative to Q1 (OR, 0.59; 95% CI: 0.46, 0.76). Similarly, in the fully adjusted model (model III), only Q4 was correlated with the risk of CKD and reduced the risk by 41% compared with Q1 (OR 0.59; 95% CI, 0.44, 0.79, *P* for trend = 0.001). The RCS model showed a nonlinear correlation between urinary strontium and CKD prevalence (Figure 2, *P* for non-linearity <0.001). The risk of CKD declined with increasing urinary strontium, but the incidence of CKD did not decrease when urinary strontium reached 258.48 ug/L.

**Table 2:** The relationship between urinary strontium and CKD in NHANES 2011–2016.

Urinary strontium categories	OR (95% CI)		
	Model I	Model II	Model III
Quartile 1	Reference	Reference	Reference
Quartile 2	0.89 (0.66, 1.19)	0.78 (0.54, 1.12)	0.81 (0.55, 1.20)
Quartile 3	0.70 (0.50, 0.96)*	0.68 (0.46, 1.01)	0.71 (0.46, 1.12)
Quartile 4	0.54 (0.43, 0.69)***	0.59 (0.46, 0.76)***	0.59 (0.44, 0.79)**
*P* for trend	<0.001	<0.001	0.001

*p < 0.05, **p < 0.01, and ***p < 0.001. OR, odds ratio; 95% CI, 95% confidence interval; CKD, chronic kidney disease; NHANES, National Health and Nutrition Examination Surveys. Model I was the crude model. Model II adjusted for age, education level, family poverty-to-income ratio, smoking status, hypertension, diabetes, anemia, serum calcium, and phosphorus. Model III adjusted for age, sex, race/ethnicity, BMI, education level, marital status, family poverty-to-income ratio, smoking status, alcohol consumption, physical activity, hypertension, diabetes, hyperlipidemia, anemia, hyperuricemia, serum calcium, and phosphorus.

### 3.3. Urinary strontium in relation to kidney biomarkers

Table 3 presents the correlation between urinary strontium and kidney biomarkers (eGFR and UACR) using the survey-weighted multivariable linear regression analysis. After adjustment for all covariables, a positive correlation was obtained between urinary strontium and eGFR [*β* (95% CI): 2.50 (0.56, 4.45) for Q2; *β* (95% CI): 2.24 (0.27, 4.21) for Q3; *β* (95% CI): 2.54 (0.32, 4.76) for Q4, *P* for trend = 0.033]. In contrast, the multivariate analysis revealed an inverse association between urinary strontium and log_2_UACR. The *β* (95% CIs) for Log_2_UACR across the strontium quartiles (Q2–Q4) were −0.13 (−0.29, 0.04), −0.23 (−0.42, −0.05), and −0.21 (−0.36, −0.05) (*P* for trend = 0.007), when compared to the Q1 group (Table 3).

**Table 3:** Multivariate linear regression analysis of urinary strontium in relation to kidney biomarkers.

Urinary strontium	eGFR		Log_2_UACR	
	Unadjusted	Adjusted	Unadjusted	Adjusted
Quartile 1	Reference	Reference	Reference	Reference
Quartile 2	2.03 (−0.26, 4.31)	2.50 (0.56, 4.45)*	−0.13 (−0.29, 0.02)	−0.13 (−0.29, 0.04)
Quartile 3	2.96 (0.60, 5.32)*	2.24 (0.27, 4.21)*	−0.32 (−0.48, −0.15)**	−0.23 (−0.42, −0.05)*
Quartile 4	4.34 (2.25, 6.43)*	2.54 (0.32, 4.76)*	−0.33 (−0.46, −0.19)***	−0.21 (−0.36, −0.05)*
*p* for trend	<0.001	0.033	<0.001	0.007

Data are expressed as the *β* and its 95% confidence intervals. 95% CI, 95% confidence interval; eGFR, estimated glomerular filtration rate; UACR, urinary albumin-to-creatinine ratio. Multivariate linear regression analysis adjusted for age, sex, race/ethnicity, BMI, education level, marital status, family poverty-to-income ratio, smoking status, alcohol consumption, physical activity, hypertension, diabetes, hyperlipidemia, anemia, andhyperuricemia, serum calcium, and phosphorus.

### 3.4. Subgroup analyses for the association between urinary strontium and CKD

**Figure 3:**
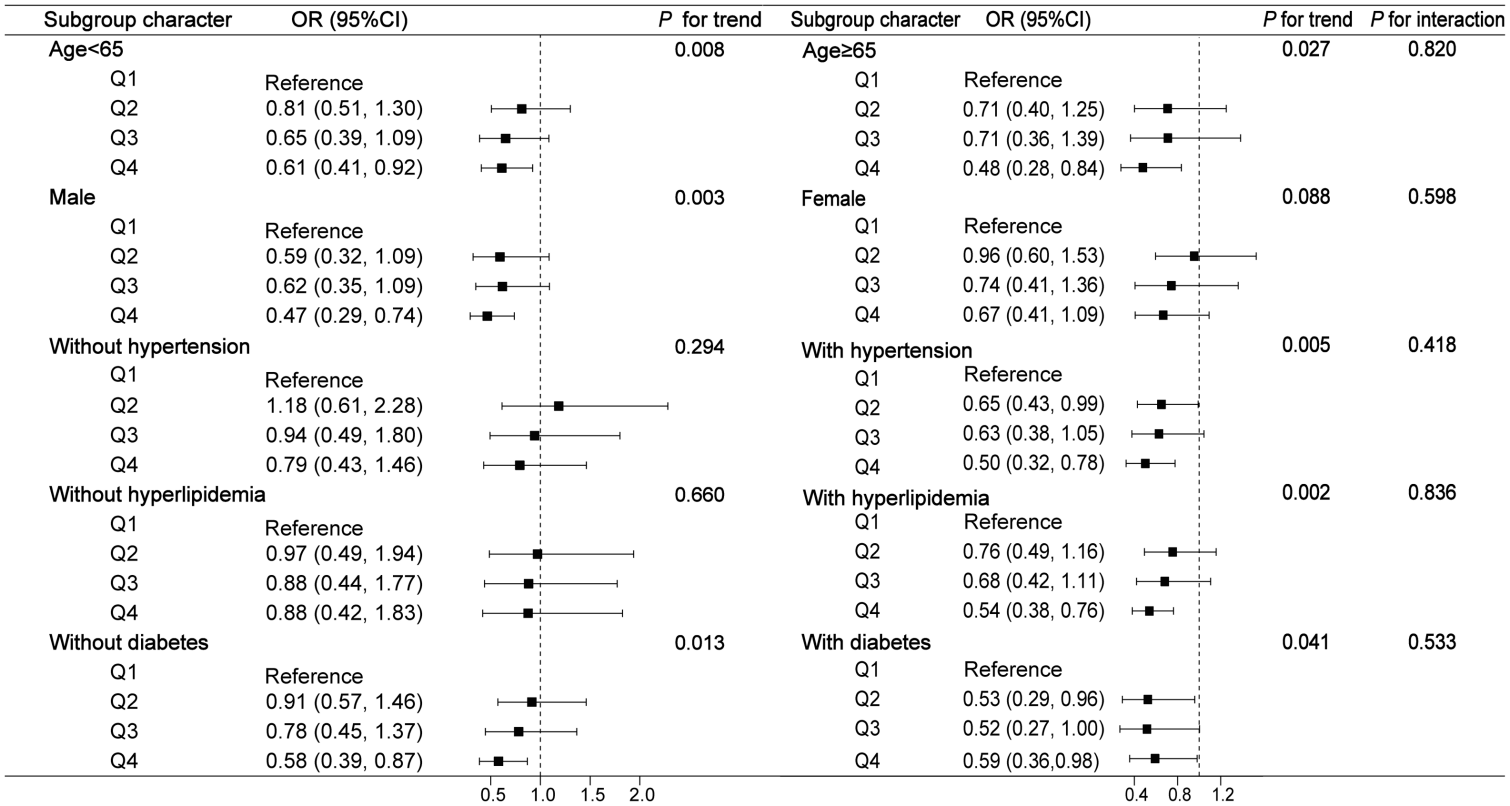
Subgroup analysis for the association between urinary strontium and CKD. OR, odds ratio; 95% CI, 95% confidence interval; CKD, chronic kidney disease.

To assess whether different subgroups modified the correlation of urinary strontium and the prevalence of CKD, we conducted stratified analyses. Whether age <65 or age ≥65, female or male, with or without hypertension, diabetes, and hyperlipidemia did not show any effect on the correlation of urinary strontium and CKD (*P* for interaction >0.05, Figure 3). The stratified model of age and diabetes indicated that the trend was statistically significant as the urinary strontium increased and the risk of CKD decreased (all *P* for trend <0.05). Moreover, participants who were male, or had a history of hypertension and hyperlipidemia exhibited a lower risk of CKD in the Q4 of urinary strontium compared to those in the Q1 (all *P* for trend <0.05).

## 4. Discussion

In this large-scale cross-sectional study targeting the nationally representative US, our analysis revealed a significant negative correlation between urinary strontium and CKD. The results also indicated that urinary strontium was positively related to eGFR but negatively to UACR. Furthermore, the risk of CKD decreased nonlinearly with the increase of urinary strontium. These findings may have positive implications for understanding the impact of strontium on kidney disease.

Strontium is abundant and widely distributed in all parts of the environment, including the earth’s crust, natural water, and human tissues (34). Strontium consumption through food is of no concern without evidence of strontium toxicity (35). Nevertheless, it has previously been demonstrated that a high dietary intake of strontium may increase the risk of bone disease, CKD, and even the need for renal replacement therapy (36–38). Most previous studies focused on the biological mechanism of plasma or serum strontium and kidney diseases and found that serum strontium was correlated with a greater risk of CKD (13–15). Considering the short biological half-life of strontium and its excretion via the kidney, urine was a non-invasive and accessible material for monitoring strontium exposure in the general population (35). There are currently no extensive reports on CKD and urinary strontium in the US general population, with only two study indicating a positive relationship between urinary strontium and eGFR (15, 17). Our analysis affirms and expands on this prior study. We found a positive correlation between urinary strontium and eGFR and a negative correlation with UACR. Importantly, our results indicated that urinary strontium was inversely associated with the occurrence of CKD. There were few specific mechanism regarding the impact of strontium on the kidneys. Previous studies found that pre-dialysis and hemodialysis patients were at risk of serum strontium accumulation (11, 12). Strontium was excreted primarily via the kidney, and a decrease in eGFR has been correlated with reduced urinary strontium (15, 17). We hypothesize that renal dysfunction may inhibit the excretion of strontium, leading to a decrease in urine strontium and an increase in serum strontium. An investigation has proposed that elevated serum strontium levels may aggravate acute kidney injury following cardiopulmonary bypass (16). While definitive conclusions cannot be drawn, it is plausible to speculate that the effects of strontium on kidney disease are mutual. Specifically, high serum strontium leads to impaired renal function, and impairment of kidney function, in turn, inhibits the excretion of strontium in urine, which explained our results observed an inverse association between urine strontium and the risk of CKD, as well as the positive correlation between serum strontium and CKD shown in previous studies (13, 14).

Most published data demonstrated a correlation between strontium levels and an increased risk of kidney events. However, no study evaluated the existence of a nonlinear relationship between urinary strontium and CKD events. RCS suggested a statistically significant L-shaped association between urinary strontium levels and CKD. The downward trend in the risk of CKD was observed with the rise in urinary strontium levels. However, the incidence rate of CKD did not exhibit a decline once the urinary strontium concentration surpassed 258.48 ug/L. This phenomenon can potentially be explained by the saturation effect.

As we all know, renal function gradually decreases with age (39), and hypertension and diabetes are the main causes of CKD. Asghari et al. demonstrated that a high-fat diet in the West Asian population could increase the incidence of CKD (40). Another study showed that participants with high-fat dietary patterns had a 49% increased rate of developing CKD, independent of diabetes and hypertension (41). Our subgroup analysis demonstrated that the association between urinary strontium and CKD was not affected by age, hypertension, hyperlipidemia, and diabetes.

The strengths of this study encompass the utilization of a nationally representative sample of US adults and a relatively large sample size, thereby enabling the generalizability of our findings. Additionally, the incorporation of comprehensive covariates, including sociodemographic characteristics, lifestyle behaviors, risk factors for CKD, and laboratory indicators, permits the control of various potential confounding factors. Furthermore, the implementation of rigorous statistical methods, such as survey-weighted multivariable logistic regression analyses and linear regression analysis, enhances the reliability of our results. Our study still has several shortcomings. First, due to the cross-sectional nature of the NHANES study, it is not possible to definitively establish a causal association between urinary strontium and CKD. Furthermore, the intricate mechanisms underlying the impact of urinary strontium on CKD remain incompletely comprehended. Second, the use of a single urine sample may not efficiently reflect chronic exposure among individuals, thereby resulting in an underestimation or overestimation of the association between urinary strontium and CKD. Additionally, failure to repeatedly measure urinary strontium may affect the precision of the estimated correlation with CKD. Finally, despite our efforts to account for a comprehensive range of potential confounding variables, unmeasured confounders may also play a role.

## 5. Conclusion

Our cross-sectional study based on US nationwide population verified a reverse and nonlinear relation between urinary strontium and CKD incidence. The results of our study offer additional perspectives on the influence of strontium on renal disease. Considering the pervasiveness of strontium in our surroundings, it is critical to fully understand its potential health implications. A large prospective cohort study with multiple assessments is required to confirm the causal relationship between urinary strontium and CKD.

## Data availability statement

The data for this study is publicly available via the NHANES website https://www.cdc.gov/nchs/nhanes/index.htm. Any queries should be directed towards the corresponding author(s).

## Ethics statement

The studies involving humans were approved by Approval was acquired from the Research Ethics Review Board of the National Center for Health Statistics. The studies were conducted in accordance with the local legislation and institutional requirements. The participants provided their written informed consent to participate in this study.

## Author contributions

FZ and NH contributed to analyzing data, interpreting results, as well as creating tables and figures. JL, MP, XL, and YL were responsible for extracting data. DL was responsible for designing the protocol, writing the paper, updating reference lists, and provided feedback on the report. All authors contributed to the article and approved the submitted version.

## Funding

This study was supported by the Science & Technology Department of Sichuan Province (2023NSFSC0603).

## Conflict of interest

The authors declare that the research was conducted in the absence of any commercial or financial relationships that could be construed as a potential conflict of interest.

## Publisher’s note

All claims expressed in this article are solely those of the authors and do not necessarily represent those of their affiliated organizations, or those of the publisher, the editors and the reviewers. Any product that may be evaluated in this article, or claim that may be made by its manufacturer, is not guaranteed or endorsed by the publisher.
